# Polarization of Cancer-Associated Macrophages Maneuver Neoplastic Attributes of Pancreatic Ductal Adenocarcinoma

**DOI:** 10.3390/cancers15133507

**Published:** 2023-07-05

**Authors:** Huey-Jen Lin, Yingguang Liu, Kailey Caroland, Jiayuh Lin

**Affiliations:** 1 Department of Medical & Molecular Sciences, University of Delaware, Willard Hall Education Building, 16 West Main Street, Newark, DE 19716, USA; 2 Department of Molecular and Cellular Sciences, College of Osteopathic Medicine, Liberty University, 306 Liberty View Lane, Lynchburg, VA 24502, USA; yliu@liberty.edu; 3 Department of Biochemistry and Molecular Biology, Molecular Medicine Graduate Program, Greenebaum Comprehensive Cancer Center, School of Medicine, University of Maryland, 108 N. Greene Street, Baltimore, MD 21201, USA; kcaroland@som.umaryland.edu (K.C.); jlin@som.umaryland.edu (J.L.)

**Keywords:** angiogenesis, chemoresistance, epithelial–mesenchymal transition, extracellular matrix, gemcitabine, hypoxia, immune checkpoint blockades, immune suppression, Kras, metastasis, pancreatic ductal adenocarcinoma, tumor-associated macrophages, tumor-infiltrating lymphocytes, tumor microenvironment

## Abstract

**Simple Summary:**

Pancreatic cancer ranks as the fourth leading cause of cancer-related death in the United States. In fact, it is estimated that there will be 64,050 new cases and 50,550 deaths in 2023 in the US alone. Pancreatic ductal adenocarcinoma accounts for the vast majority of pancreatic cancer cases, and it has been widely recognized as one of the most devastating malignancies. The majority of patients are diagnosed at late stages when metastasis has occurred, leading to the 5-year survival rate being below 10%, which is the lowest among all cancer types. The causes of death are largely attributed to scanty screening diagnostic tools, abrupt metastasis, and prevalent chemoresistance. Molecular studies have elucidated that the stiff fibroblastic stroma shields from the penetration of therapeutic agents and establishes a hypoxic niche. A growing body of evidence identifies that tumor-associated macrophages play pivotal roles contributing to mortality by strengthening the fibroblastic stroma, promoting malignant cell proliferation, augmenting angiogenesis, metastasis, acquiring pleiotropic pancreatic cancer stem-like cells, supporting chemoresistance, and harnessing an immune-suppressive microenvironment that subsequently dampens chemo- and immunotherapies. This review will summarize research findings revealing various mechanisms employed to polarize macrophages to tumor-supporting subtypes which subsequently unleash the plethora of neoplastic characteristics. In addition, it will ignite potential targets aiming to correct the aberrant carcinogenic regulators through therapeutic approaches.

**Abstract:**

Mounting evidence links the phenomenon of enhanced recruitment of tumor-associated macrophages towards cancer bulks to neoplastic growth, invasion, metastasis, immune escape, matrix remodeling, and therapeutic resistance. In the context of cancer progression, naïve macrophages are polarized into M1 or M2 subtypes according to their differentiation status, gene signatures, and functional roles. While the former render proinflammatory and anticancer effects, the latter subpopulation elicits an opposite impact on pancreatic ductal adenocarcinoma. M2 macrophages have gained increasing attention as they are largely responsible for molding an immune-suppressive landscape. Through positive feedback circuits involving a paracrine manner, M2 macrophages can be amplified by and synergized with neighboring neoplastic cells, fibroblasts, endothelial cells, and non-cell autonomous constituents in the microenvironmental niche to promote an advanced disease state. This review delineates the molecular cues expanding M2 populations that subsequently convey notorious clinical outcomes. Future therapeutic regimens shall comprise protocols attempting to abolish environmental niches favoring M2 polarization; weaken cancer growth typically assisted by M2; promote the recruitment of tumoricidal CD8^+^ T lymphocytes and dendritic cells; and boost susceptibility towards gemcitabine as well as other chemotherapeutic agents.

## 1. Introduction

### 1.1. Introduction of Pancreatic Cancer

Pancreatic carcinoma is the deadliest malignancy afflicting the exocrine digestive organ. This cancer is well known for lacking screening tools and having early metastatic spread, followed by chemoresistance, leading to limited treatment strategies and poor prognostic outcomes [1,2]. As such, it took 466,003 lives across 185 countries in 2020 and is presently the seventh leading cause of deaths from cancers in both genders [3]. Trends forecasted through 2040 predict that pancreatic cancer will become the second-most-leading cause of cancer-related death in the United States [4], and approximately 355,317 new cases will occur globally [5]. Among them, nearly 95% of pancreatic cancer incidences are pancreatic ductal adenocarcinoma (PDAC) [6]. Approximately 80% of pancreatic cancer patients present with advanced-to-late stages of nonresectable and disseminated disease [7]. The two most common first-line chemotherapeutic regimens include blends of 5-fluorouracil, leucovorin, irinotecan, and oxaliplatin (FOLFIRINOX) and gemcitabine (GEM) plus Nab-Paclitaxel [8]. However, therapeutic intervention scarcely improves overall prognosis, and the 5-year survival rate remains disappointing [9,10].

PDAC develops sporadically but is largely due to the acquisition of constitutively active mutant Kirsten rat sarcoma (KRAS) derived from the most frequent driver mutations: G12R, G12V, and G12D, which comprise approximately 90% of occurrence [11]. Among them, G12D accounts for about 40% of incidents [11,12]. Initial progression to pancreatic cancer embarks from the cells harboring KRAS mutations engaging in networking with proinflammatory cytokines [13,14,15]. For instance, in response to oncogenic mutant KRAS, interleukin (IL)-6 induces the expression and activation of signal transducer and activator of transcription 3 (STAT3) [15,16,17,18]. Accordingly, persistent STAT3 signaling was demonstrated to play a pivotal role in mutant KRAS-induced pancreatic tumorigenesis [19], and demonstrated that Janus kinase (JAK)–STAT3 axis activation correlates with a poor outcome in PDAC patients following surgical resections [20]. Moreover, oncogenic mutant KRAS unleashes a plethora of signaling cascades, including rapidly accelerated fibrosarcoma (RAF)/mitogen-activated protein kinase (MEK)/extracellular signal-regulated kinase (ERK), and phosphoinositol 3-kinase (PI3K)/protein kinase B (AKT) pathways in various malignant entities including pancreatic cancer [11,21,22,23]. RAF/MEK/ERK is the first well-known Ras effector in cancers. GTP-bound KRAS interacts with and triggers RAF, which further induces the phosphorylation and activation of MEK1 and MEK2. This scenario subsequently enhances ERK1 and ERK2 serine/threonine kinases activities. Activated ERK1/2 then phosphorylates over 200 targets, many of which are transcription factors controlling cell proliferation [24,25]. Mounting evidence demonstrates the critical role of PI3K being a regulator for embarking oncogenic KRAS-driven carcinogenesis, largely by governing cell survival and proliferation [26,27]. Another independent study utilizing a genetically engineered mouse model containing mutant *Kras* elucidates a similar finding that the PI3K pathway can augment PDAC through the activation of STAT3 and nuclear factor kappa B (NF-κB) signaling [28].

The first histological alteration occurring in PDAC pathogenesis is the transdifferentiation of acinar cells into duct-like cells, named acinar-to-ductal metaplasia (ADM) [29,30]. The molecular causes underlying dysregulated ADM were recently elucidated to be associated with a loss of AT-rich interactive domain containing protein 1A (ARID1A) [31], followed by interaction between PAF1 (RNA polymerase II-associated factor 1) and YAP1 (yes activated protein-1) [32]. For ADM, infiltrating macrophages secrete inflammatory cytokines including regulated on activation normal T cell expressed and secreted (RANTES) [33] and tumor necrosis factor-alpha (TNF-α). Together, they lead to the activation of NF-κB signaling and expression of matrix metalloproteinases (MMPs) [33,34]. In response to chronic inflammation, acinar pancreatic cells adopt ADM [29] and then develop precancerous lesions, which are not only frequently observed in pancreatitis [35], but also develop into pancreatic intraepithelial neoplasia (PanIN) following the acquisition of oncogenic mutations such as KRAS [29]. Both ADM and PanIN constitute crucial aberrations in PDAC and persist throughout tumor development [34,36]. During this neoplastic progression, macrophage depletion not only blocks the progression of ADM to PanIN, but also lightens PDAC burden in mice [34,37], underscoring the imperative role played by these immune cells.

Although oncogenic *Kras* mutation in mouse PDAC is critical for cancer initiation, constitutively activated mutant KRAS alone is insufficient for tumor onset; rather, it requires partner mutations such as the *P53* tumor suppressor gene, as well as cytokines produced by different cell types within the tumor mass [38]. A genetically engineered mouse model combining both mutations, *LSL-KrasG12D*; *Trp53flox/flox*; *Pdx-1-Cre* (KPC), has been established as a clinically relevant PDAC model that recapitulates many key features of human PDAC with a robust inflammatory response [39] and elevated immunosuppressive features [40].

### 1.2. Introduction of Tumor Microenvironment and Immune Evasion

Marked by extensive fibrosis and inflammation, PDAC’s tumor microenvironment (TME) consists of fibroblasts, immune cells, endothelial cells, and an acellular extracellular matrix (ECM) that contains various growth factors, chemokines, and cytokines [41]. Within the TME, cancer cells interplay with nearby stroma and acellular constituents that synergistically controls malignant traits and therapeutic outcomes [42,43]. Fibroblastic stroma can hinder drug entry by safeguarding tumor cells from therapeutic insults [44], and then advancing tumor progression characterized by invasion, angiogenesis, metastasis, and chemoresistance [45]. PDAC is initially featured with chronic inflammation triggered by immune aberrations [46]. Then, oncogenic mutant KRAS augments inflammation and launches an immunosuppressive TME that subsequently plays a pivotal role in cancer progression [47,48,49,50].

In general, immune responses are modulated by a plethora of checkpoint regulators that act as “security brakes” and establish a “do not eat me” cue when inflammation reactions shall be ended from prior infections, or autoimmunity shall be circumvented by enhancing self-tolerance. Cancers exploit various immune checkpoint modulators, attempting to evade tumoricidal responses, favor immune tolerance, and escape recognition and clearance by immune surveillance cells [51]. Therapeutic agents abolishing such functions are recognized as immune checkpoint blockades (ICBs) that have been proven to improve clinical outcomes [52]. Yet, PDAC remains largely embraced by an immunosuppressive TME with limited infiltration of tumoricidal immune cells, thereby resulting in a poor response to ICBs [53,54]. The TME attracts several immunosuppressive cell types that circumvent the surveillance normally conducted by cytotoxic cluster of differentiated (CD)8^+^ T lymphocytes and by dendritic cells (DC) [53].

Within the TME of PDAC, infiltration of tumoricidal CD8^+^ T lymphocytes is rare. Accordingly, a few well-known ICBs attempting to revive T lymphocytes to date have manifested disappointing efficacy [55]. Instead, the tumor bed is infiltrated with largely protumorigenic immune-suppressive cells including myeloid-derived suppressor cells (MDSC), regulatory T cells (Treg), and tumor-associated macrophages (TAM) [47,53]. TAMs are the earliest infiltrating cells in PanIN lesions and continue to rise throughout cancer progression [56]. Macrophages in PDAC are derived from blends of circulating monocytes and phagocytes that reside in the pancreas. Moreover, TAMs are the most abundant immune cells in the stroma and are the key drivers shaping the immunosuppressive landscape [57]. TAMs enhance tumor immune evasion, mainly by enhancing tumor fibrosis and excluding tumoricidal T lymphocytes [58]. TAM infiltration not only correlates with lymph node metastasis and poor prognosis [59], but also plays multifaceted roles in the carcinogenesis of PDAC [60].

As a vital innate immune population for maintaining body homeostasis and warding off foreign particles or pathogens, macrophages can regularly sense their microenvironment, display high plasticity, and execute diverse functions adapted to different environmental contexts. Depending on the inflammatory cues, macrophages can develop two distinct subtypes, these being either classically activated M1 or alternatively activated M2 subpopulations [61]. M1 macrophages are proinflammatory and tumoricidal, whereas M2 macrophages are anti-inflammatory, protumorigenic, and immunosuppressive [61,62]. Furthermore, fully polarized macrophages can depolarize and transform reciprocally in response to environmental triggers [63]. The M1 subtype commonly produces higher levels of IL-1, IL-6, IL-12, IL-23, TNFα, chemokine C-X-C motif ligand (CXCL)9, CXCL10, and inducible nitric oxide synthase (iNOS) [64]. Conversely, the M2-type commonly expresses higher levels of IL-10, transforming growth factor-β1 (TGF-β1), and arginase 1 (ARG1) [65,66,67,68,69,70]. M2 is the most abundant immunosuppressive subpopulation representing approximately 85% of TAMs [53,57,71,72]. Infiltration and the abundance of M2 is not only a malignant hallmark but also correlates with poor prognosis [73,74]. Yang et al. demonstrated that targeting proliferating F4/80+ macrophages by the pharmacological inhibitor, clodronate liposomes, fostered CD8^+^ T cell infiltration and promoted their spatial redistribution, thereby enhancing antitumor immunity [75]. Furthermore, closer proximity of M2 macrophages to the tumor core strongly correlates with poor disease-free survival [69], highlighting the clinical impact of M2 macrophages on molding a cancer-promoting landscape [61,71,76].

Macrophages exist on a spectrum of polarization states between the M1 and M2 phenotypic extremes and exhibit functional plasticity within the TME [77]. The early stages of tumor lesions initially have a high abundance of M1 macrophages that are later polarized to the M2 population as PDAC progresses [78]. Preclinical and clinical trials have been completed, or are still ongoing, attempting to target TAMs and treat various cancer types including pancreatic cancer (e.g., NCT03662412, NCT03184870, and NCT01921699) [79]. Although M2 macrophages are still under substantive studies, this report aims to extrapolate PDAC-fostered M2 macrophages, delineate TME-orchestrated mechanisms responsible for M2 polarization, and then discern how the M2 population synergizes cancer cells and TME factors to convey multifaceted impacts on PDAC. Due to space limitations, the authors regret that some outstanding findings cannot be discussed in this article.

## 2. Factors Modulate Polarization of TAM

### 2.1. Factors Released from Malignant Cells or Cancer-Associated Fibroblasts (CAFs)

**Figure 1:**
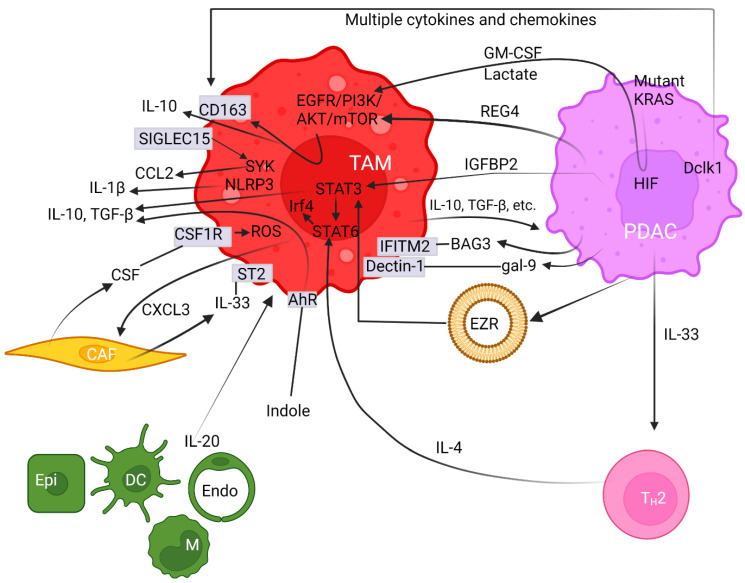
Pancreatic ductal adenocarcinoma cells synergize with the tumor microenvironment to provoke polarization of M2 macrophages. Arrows with pointed or with blocked ends indicate activation or inhibition between regulators, respectively, while a fading effect at the start of arrows represents secretion of modulators. Plain straight lines depict interaction between ligands and receptors. Cell surface proteins are noted in rectangular boxes on cell membranes. The circular lipid bilayer depicts an extracellular vesicle. Abbreviations used include aryl hydrocarbon receptor (AhR), protein kinases B (AKT), Bcl-2-associated athanogene 3 (BAG3), cancer-associated fibroblast (CAF), CC-chemokine ligand (CCL), cluster of differentiated (CD), CXC chemokine ligand (CXCL), colony-stimulating factor (CSF) and receptor (CSF1R), dendritic cell (DC), double cortin-like kinase 1 (Dclk1), endothelial cell (Endo), epidermal growth factor receptor (EGFR), epithelial cell (Epi), ezrin (EZR), galectin (gal), granulocyte-macrophage colony-stimulating factor (GM-CSF), hypoxia-inducible factor (HIF), interleukin (IL), insulin-like growth factor binding protein 2 (IGFBP2), interferon-induced transmembrane protein 2 (IFITM-2), interferon regulatory factor 4 (Irf4), Kirsten rat sarcoma (KRAS), mammalian target of rapamycin (mTOR), microRNA (miR), monocyte (M), nucleotide-binding and leucine-rich repeat receptor containing pyrin domain 3 (NLRP3), pancreatic ductal adenocarcinoma (PDAC), phosphatidylinositol 3-kinase (PI3K), reactive oxygen species (ROS), regenerating gene family member 4 (REG4), sialic-acid-binding immunoglobulin-like lectin 15 (SIGLEC15), signal transducer and activator of transcription (STAT), spleen tyrosine kinase (SYK), suppression of tumorigenicity 2 (ST2), tumor-associated macrophage (TAM), transforming growth factor β (TGF-β), and T helper-2 (T_H_2).

Crosstalk between neoplastic cells and infiltrating macrophages in the tumor milieu governs PDAC carcinogenesis. TAMs are in close contact with cancer-secreted factors and thereby are polarized towards the M2 phenotype [37,80,81]. Intriguingly, oncogenic mutant KRAS can recruit TAMs and then promote carcinogenesis [80]. Mutant KRAS not only releases growth factors but also regulates glucose metabolism in PDAC [82]. Accordingly, lactate and granulocyte-macrophage colony-stimulating factor (GM-CSF) are known to be profoundly released from cancer cells expressing oncogenic mutant KRAS [82,83] (Figure 1). This aberration is mediated through the PI3K/AKT signaling cascade that partly enhances macrophage polarization [84]. Moreover, regenerating gene family member 4 (REG4) released from PDAC cancer cells [85,86] can promote macrophage polarization to M2, as well as orchestrate the TME to favor cancer growth and metastasis [87] (Figure 1). Consequently, high numbers of M2-polarized TAMs correlate with an increased incidence of lymph node metastasis [87]. The underlying molecular mechanism accounting for this scenario was deemed to be mediated through the epidermal growth factor receptor (EGFR)/AKT/cAMP-response element binding protein (CREB) signaling pathway [87]. A further study elucidated that the overexpression of recombinant REG4 enhanced the expression of IL-10, CD163, and many other M2 signature genes in TAMs [87]. Additionally, the secretion of IL-10 can be upregulated by insulin-like growth factor binding protein 2 (IGFBP2) released from cancer cells following STAT3 activation [88] (Figure 1). IGFBP2 favors M2 macrophages and exacerbates an immunosuppressive TME by increasing Treg infiltration and inhibiting antitumor T cell immunity in a mouse model [88]. Hence, blocking the IGFBP2 axis constitutes a promising treatment protocol through which TAM polarization can be attenuated and a tumoricidal state of the TME can be revived [88]. Together, multiple networks maneuver TAM polarization toward an M2 state.

Double cortin-like kinase 1 (Dclk1) is overexpressed in the cancer cores and PanIN lesions, based off various pancreatic cancer models [89] (Figure 1). By releasing various chemokines and cytokines, the elevated Dclk1-isoform 2 resulted in the polarization towards the M2 phenotype (Figure 1). This aberration is demarcated by a high abundance of M2 macrophages and low occupancy of CD8^+^ T cell infiltration with weakened tumoricidal activities [90]. These M2 macrophages enhance cell migration, invasion, and self-renewal, along with increased expression of Snail and Slug, both of which are indicatives of cancer stem-like cells [90,91]. Moreover, galectin-9 (gal-9), a member of the P-galactoside-binding family of lectins, was found to be highly expressed in both mouse and human PDAC. The binding of gal-9 to its receptor, Dectin-1, a crucial innate immune regulator expressed on the surface of macrophages, polarizes macrophages to the M2 phenotype (Figure 1). Disruption of the gal-9/dectin-1 interaction reverts immunosuppression, enhances cytotoxic T lymphocytes recruitment, downregulates Tregs, impedes tumor growth, and achieves improved therapeutic efficacy [92,93,94]. Moreover, Ezrin (EZR) expression is upregulated in PDAC and is associated with tumor progression [95]. Chang et al. demonstrated that extracellular vesicles (EVs)-capsulated EZR is strikingly correlated with poor survival in PDAC patients [96]. Molecular investigations further discerned that overex pressed EZR regulates STAT3 activation [97] that further synergizes with STAT6 to augment the polarization of TAMs towards the M2 phenotypes [98] (Figure 1). Consistently, Su et al. reported miR-155 and miR-125b2 as being the key regulator encapsulated in the PDAC cell-line-derived EV that exploits a dose-dependent effect on macrophage plasticity [99].

On the other hand, CAFs release colony-stimulating factor (CSF) and induce M2 polarization through binding to receptor CSF1R within the PDAC milieu, and then enhance reactive oxygen species (ROS) production in monocytes [100] (Figure 1). The importance of ROS activation on M2 polarization was illustrated by the evidence that ROS ablation abrogates this effect [101]. Anti-CSF1R therapy favors the M1-like subpopulation in vivo, thereby exerting a powerful antitumor effect on glioma neoplasm [102]. Furthermore, stromal fibroblasts are the predominant cell types for producing IL-33 that mainly targets its receptor, known as suppression of tumorigenicity 2 (ST2), on TAMs and induces the polarization of M2 [103,104]. Upon activation, IL-33-polarized TAMs subsequently release CXCL3 to further amplify CAFs. Together, this interactive axis constitutes a paracrine and positive feedback loop amplifying both CAF and TAM cell types [105] (Figure 1).

### 2.2. Factors Produced from Stromal Immune Cells

Abundantly in PDAC, oncogenic mutant Kras can activate the downstream PI3K/AKT/mammalian target of the rapamycin (mTOR) signaling pathway [106]. Consequently, the aberrant activation of this cascade conveys tumor initiation, cancer progression, and metastatic spread, followed by emerging chemoresistance [107]. This signaling axis can be effectively abrogated by urolithin A (Uro A) [108]. The treatment of PDAC cells with Uro A not only inhibited the growth of tumor xenografts and improved the overall survival (OS) of *Ptf1aCre/+;LSL-KrasG12D/+;Tgfbr2flox/flox* (PKT) mice, but also reprogrammed the tumor microenvironment by attenuating infiltrated immunosuppressive cells such as TAMs, MDSCs, and Tregs [108].

Oncogenic mutant Kras^G12D^ elevates IL-33 expression in PDAC cells, which recruits and activates T_H_2 cells. Then, T_H_2 cells stimulate tumor growth by secreting protumorigenic cytokines such as IL-4 that exerts major impacts on neighboring innate immune cells (Figure 1). Studies on animal models unveiled that IL-4-initiated signaling in macrophages can be further orchestrated by Stat6, which in turn regulates interferon regulatory factor 4 (Irf4) that acts as an important transcription factor and harnesses M2 polarization [109] (Figure 1). Conversely, Irf4 deficiency impeded the expression of M2-associated signature genes [110]. In a syngeneic model of PDAC, the inhibition of Irf4 using the immunomodulatory agent pomalidomide resulted in a shift of macrophages towards the M1 population and fosters an immune surveillance antitumor environment along with an improved infiltration of cytotoxic T lymphocytes and enhanced immune responses [111].

Proinflammatory cytokine IL-20 is a member of the IL-10 family and is expressed predominantly by epithelial cells, monocytes, dendritic cells, and endothelial cells in the TME (Figure 1). IL-20 was demonstrated to promote M2 polarization, and elevated IL-20 levels in PDAC tumor tissue correlate with poor overall survival [112]. Inhibiting IL-20 using an antagonistic antibody, 7E, reshapes the TME toward scenarios unfavorable for malignancies in multiple aspects including diminished M2 macrophage infiltration, lightened fibrosis, inhibited tumor growth, and reduced expression of the immunosuppressive regulator PD-L1 on tumor cells [112].

TAMs remain the primary cell type molding the immune landscape [75,113], partly fortified by a self-amplifying mechanism. Sialic-acid-binding immunoglobulin-like lectin 15 (SIGLEC15) is upregulated in M2 macrophages and could directly enact immunosuppressive function via binding α-2,3 sialic acid [114]. Stimulation of the extracellular domain of SIGLEC15 promotes the tyrosine phosphorylation of DNAX-activating protein of 12 kDa (DAP12) and leads to the activation and recruitment of spleen tyrosine kinase (SYK) [115] (Figure 1). Joshi et al. further revealed an autocrine-positive feedback loop phenomenon by demonstrating that SYK, in conjunction with the PI3K axis, synergizes M2 polarization, which can be abolished by a dual SYK/PI3K inhibitor, SRX3207 [116]. α-2,3 sialic-acid-bound SIGLEC15 enhances the production of C-C motif chemokine ligand (CCL)2, C-X-C motif chemokine (CXCL)2, and CXCL8 in TAMs, which not only exacerbates immune suppression but also accelerates tumor progression in gastric [117], esophageal [118], and bladder carcinomas [119]. Among them, CCL2 facilitates the mobilization of receptor CCR2^+^ inflammatory monocytes from bone marrow to the tumor bed, where they become immunosuppressive TAMs [120]. Together, SIGLEC15 expression, monocyte mobilization, and M2 polarization form a positive feedback circuit, enabling the recruitment and amplification of TAMs [114]. In this regard, a clinical trial in patients with nonmetastatic PDAC using the orally dosed small-molecule CCR2 inhibitor (CCR2i) PF-04136309, in combination with FOLFIRINOX, demonstrated improved antitumor efficacy (trial number NCT01413022). However, a compensatory influx of CXCR2^+^ neutrophils resulted in a relapse. Yet, this therapeutic resistance can be circumvented by combinatorial blockades targeting both types of infiltrating myeloid cells. Dual treatments not only promote a robust antitumor effect compared to either inhibitor alone, but also improve the overall response to FOLFIRINOX [121].

On the other hand, CD40, a cell surface receptor belonging to the TNF superfamily, can regulate myeloid cell function and adaptive immunity. Similar to Toll-like receptors (TLRs), the CD40 pathway acts as a linkage between DCs and adaptive immunity in cancer. Ligands of CD40 (CD40L) connect DCs and other immune cells in response to malignancies or pathogenic insults with memory. Yet, agonistic anti-CD40 (αCD40) monoclonal antibodies mimic CD40L in vivo and have been shown to enhance the immunogenicity of cancer vaccines and trigger cancer regressions [122,123,124], including in pancreatic cancer [125]. Interestingly, one of the well-studied αCD40 antibodies, selicrelumab, was taken into clinical evaluation as a novel agent for immune activation and cancer immunotherapy, independent from ICB [126]. CD40 activation by selicrelumab enhanced the polarization of TAMs towards the M1 phenotype, as well as activated the proliferation and infiltration of CD8^+^ T lymphocytes and DCs [126,127]. Together, this treatment transforms the TME from “cold” to “hot” immunity [126,127]. Surgical samples from patients receiving selicrelumab preoperatively exhibited decreased tumor fibrosis, fewer M2 macrophages, and a greater maturation of intratumoral DCs [127]. It is noteworthy to mention that, clinically, combinatorial treatments using αCD40 antibodies and ICB ameliorate efficacy in patients who are initially refractory to immunotherapies. Accordingly, Winograd et al. developed an effective treatment regimen with αCD40 antibodies and ICB (αPD-1/αCTLA-4) using a genetically engineered KPC mouse model [128]. Such success exemplified that the combination of αCD40/ICB, but not either of αCD40 or ICB alone, results in a prominent decline in tumor burden and gain of immunological memory [128].

### 2.3. Aberrant Metabolism, Hypoxic TME, and Dysregulated Epigenetics

Indole compounds are evolved from dietary tryptophane upon metabolizations by gut microbials such as lactobacilli. Indoles are the key activators for aryl hydrocarbon receptor (AhR), although tryptophane metabolism by human cells rendered negligible effects. By promoting the polarization of TAMs to M2, elevated AhR expression has been recognized as a central driver of TAM function in responding to multiple cues to promote an immune-suppressive state of the TME [129] (Figure 1). Molecular studies delineate that high expression of AhR inhibits IFNγ expression in CD8^+^ T cells [129], while it enhances the expression of immunosuppressive IL-10 [130], TGF-β, and Arg1 [131,132] (Figure 1). The aforementioned data on animal models coincide with clinical facts in which patients with high AhR expression are strongly correlated with rapid disease progression and increased mortality, along with the immune-suppressive properties associated with TAMs, underscoring the conservation of this regulatory axis in PDAC [129].

PDAC ubiquitously fosters a hypoxic TME. Hypoxia is a condition where the oxygen pressure is below 5–10 mm of mercury, and this phenomenon can empower cancer metastasis [133]. The major mechanism executing cellular responses toward hypoxia is the activation and sustainment of hypoxia-inducible factors (HIFs), mainly HIF1 and HIF2, that activate a set of genes facilitating tumor growth, angiogenesis, and metastasis [134,135]. On the other hand, the endocytosis of cancer, or immune or endothelial cells, can form and release extracellular exosomes [136]. The tumor-derived exosomal miR-301a-3p, for example, not only is released from hypoxic PDAC, but also promotes M2 polarization and ameliorates the PTEN/PI3Kγ pathway, thereby enhancing metastasis in vitro and in vivo [137]. Stimulated by a hypoxic TME, HIF-1α further augments the expression of glycolytic enzymes contributing to maintaining bioenergetic homeostasis during hypoxic stress [138]. In support of this notion, inflammatory cells such as TAMs tend to maneuver metabolism toward glycolysis to meet high energetic demand [139]. Recent studies have unveiled that hypoxia and glycolysis-related gene signatures are concurrently associated with an unfavorable TME and are used to predict a poor prognosis of PDAC patients [140]. Hypoxia and glycolysis pathways are upregulated in the prognostically high-risk cohorts compared to the low-risk counterparts [141,142]. Apart from glucose metabolism, the ablation of HIF2 in CAFs modestly reduces fibrosis and significantly decreases the intratumoral recruitment of M2 macrophages and Treg cells. Similarly, treatment with the clinical HIF2 inhibitor PT2399 abolishes paracrine signaling driven by HIF2, and significantly reduces M2 polarization as well as improves tumor responses to immunotherapy using ICB in PDAC mouse models [143].

GEM treatment favors TAM infiltration into the tumor mass and shifts the stroma to a predominantly M2 phenotype that conveys notorious survival [79], owing to the destruction of gemcitabine by M2-released pyrimidines [144]. Furthermore, paracrine signals from the removal of chemotherapy-generated apoptotic cells can stimulate immune-suppressive controllers in the TME. The phagocytosis of apoptotic cells increases the production of TGF-β1, prostaglandin E2 (PGE2), and platelet-activating factor (PAF), all of which are known to act as anti-inflammatory and immune-suppressive modulators [145].

Dysregulated epigenetic modulators can influence TAM polarization. An epigenomic analysis of TAMs isolated from PDAC tissues revealed the overexpression of CCCTC binding factor (CTCF), an important epigenetic regulator in TAMs. CTCF can enhance M2 polarization and favor the tumor-promoting properties of the TAMs. CTCF-transcribed long noncoding RNA (LncRNA) of prostaglandin-endoperoxide synthase 2 (PTGS2) antisense NF-*κ*B1 complex-mediated expression regulator RNA (PACERR) can orchestrate PTGS2 expression. A novel investigation demarcated that transcribed LncRNA PACERR binds CTCF, forming the CTCF/PACERR complex to recruit the E1A binding protein p300 (EP300), which is one of the histone acetyltransferases. Being an epigenetic regulator, this complex not only enhances chromatin accessibility, but also elevates PTGS2 transcription. Excessively expressed PTGS2 is one of the key activators for polarizing M2 [146].

Moreover, cancer progression and the chemoresistance of PDACs have been associated with elevated histone deacetylases (HDACs) and glycogen synthase kinase 3 beta (GSK3B) activity. Accordingly, treatment by the dual inhibitor, metavert, lowers the abundance of M2 macrophages by more than 50%, although the total number of macrophages are unaffected significantly [147]. These data implicate the molecular cue leading to cancer inhibition by metavert is partially due to the reversion of M2 to the M1 phenotype [147]. Metavert treatment further downregulates procancer cytokines like IL-6 and IL-4, induces cancer cell apoptosis, and attenuates the expression of cancer stem cell markers, as well as impedes cancer growth and metastases [147].

## 3. Impact of M2 on Neoplastic Features of PDAC

TAM density is an independent prognostic determinant correlated with a higher risk of disease progression, recurrence, and metastasis, and shorter OS in human PDAC patients [148]. Such unequivocal evidence is recapitulated in preclinical mouse models [121,149,150]. M2 macrophages regulate a plethora of critical carcinogenic traits, including enhanced chemoresistance, cancer growth, angiogenesis, metastasis, and immune suppression [151,152].

### 3.1. TAMs Enhance Chemoresistance

**Figure 2:**
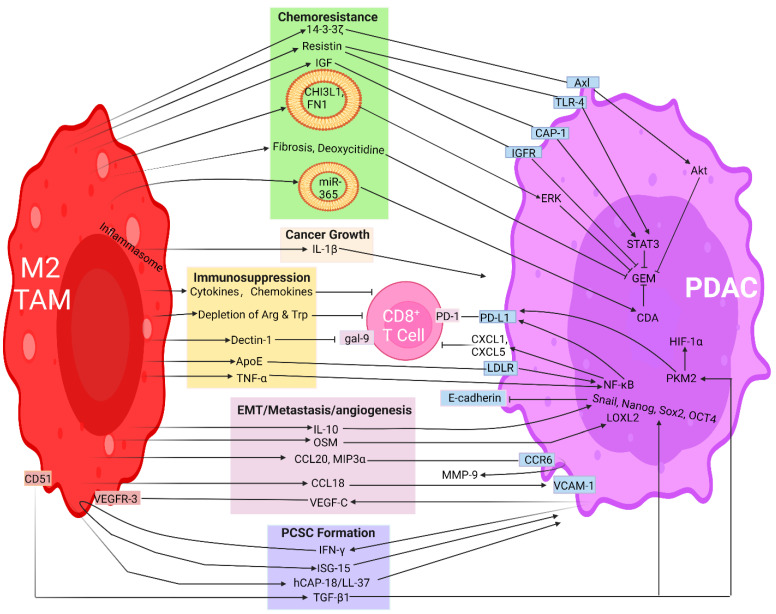
M2 macrophage promotes a plethora of neoplastic features of pancreatic ductal adenocarcinoma and suppresses tumoricidal effects exerted from cytotoxic T lymphocyte. Arrows and straight plain lines, as well as overlapping abbreviations used in both figures, are described in the legend of Figure 1. Additional abbreviations that are used in Figure 2 include apolipoprotein E (ApoE), arginine (Arg), adenylyl cyclase-associated protein 1 (CAP-1), CC-chemokine ligand (CCL), CC-chemokine receptor (CCR), cytidine deaminase (CDA), chitinase 3-like-1 (CHI3L1), CXC chemokine ligand (CXCL), endothelial cell (EC), epithelial–mesenchymal transition (EMT), extracellular signal-regulated kinase (ERK), fibronectin-1 (FN1), gemcitabine (GEM), hypoxia-inducible factor 1α (HIF-1α), interferon (IFN), insulin-like growth factor (IGF) and receptor (IGFR), IFN-stimulated gene 15 (ISG15), immunomodulatory cationic antimicrobial peptide 18/LL-37 (hCAP-18/LL-37), low-density-lipoprotein receptor (LDLR), lysyl oxidase-like protein 2 (LOXL2), macrophage inflammatory protein-3α(MIP3α), microRNA (miR), matrix metalloproteinase 9 (MMP-9), nuclear factor κ-light-chain-enhancer of activated B cells (NF-κB), oncostatin M (OSM), pancreatic cancer stem-like cells (PCSCs), programmed death receptor-1 (PD-1) and ligand (PD-L1), pyruvate kinase isoform M2 (PKM2), Toll-like receptor 4 (TLR4), tryptophan (Trp), tumor necrosis factor α (TNF-α), vascular cell adhesion molecule 1 (VCAM-1), vascular endothelial growth factor (VEGF) and receptor (VEGFR), and YWHAZ/14-3-3 protein zeta/delta (14-3-3ζ).

Desmoplastic stroma in PDAC impedes the penetration of therapeutic agents. Macrophages are well known for their ability to promote fibrosis under various physiological and pathological conditions [153]. A recent study revealed that subsets of TAMs augment fibrosis through directly depositing or remodeling the ECM [154] (Figure 2). Hence, following coculture with M2, PDAC cells demonstrate elevated fibroblastic characteristics [155]. Furthermore, TAMs promote chemoresistance against GEM therapy [156]. GEM is a synthetic cytidine analog that inhibits cell proliferation by pausing DNA replication and arresting RNA transcription. Resistance to GEM arises in weeks following treatments, owed to a combination of intrinsic resistance and adapted modulators residing in the TME [157]. GEM is typically metabolized intracellularly by deoxycytidine kinase (DCK) to an active form of phosphonucleosides. The incorporation of these nucleosides into DNA or RNA results in proliferative arrest. A growing body of evidence elucidates that GEM resistance can be attributed from a wide variety of mechanisms, including drug transporter loss, DCK deficiency, competition between endogenous cytidine triphosphate and phospho-GEM, and elevated cytidine deaminase (CDA) expression that abolishes GEM’s action mode by converting it to the inactive compound 2′,2′-difluoro-2′-deoxyuridine [158]. Treatment with nab-paclitaxel partly hinders chemoresistance by lowering CDA expression; underscoring treatments with dual agents can circumvent treatment resistance [159]. Intriguingly, TAMs can modulate therapy resistance by upregulating CDA in cancer cells [160] and by releasing pyrimidine nucleoside deoxycytidine that competes with GEM and lowers its active dose [144] (Figure 2). Hence, the depletion of proliferating TAMs using clodronate liposomes improves the therapeutic response towards GEM in a tumor-bearing mouse model [161].

Apart from lightening the GEM burden, TAMs convey drug resistance by muting signals from apoptotic cells through secreting various factors and attenuating apoptosis, thereby favoring chemoresistance [60]. Briefly, following the phagocytosis of apoptotic PDAC cells, TAMs secrete an antiapoptotic factor known as YWHAZ/14-3-3 protein zeta/delta (14-3-3ζ). Through binding to its interacting partner, the Axl receptor tyrosine kinase, this complex stimulates the phosphorylation of Akt in PDAC, activates cellular prosurvival mechanisms, and enacts a crucial regulator of antiapoptotic pathways that renders a compelling chemoresistance (Figure 2). During chemotherapy, extracellular 14-3-3ζ released from macrophages is imperative for enabling PDAC cells to combat prolonged and continuing chemotherapeutic pressure [60]. These data highlight a distinct regulatory mechanism by which chemotherapy-induced apoptosis ignites an antiapoptotic/protumor mechanism elicited by TAMs and presents a therapeutic challenge pertaining to how apoptotic death provokes paradoxical chemoresistance in PDAC.

It is worth mentioning that TAMs can promote chemoresistance by producing insulin-like growth factors (IGF)-1 and -2, which bind and activate IGF receptors on pancreatic cancer cells [162], as well as by releasing resistin, which binds to adenylyl cyclase-associated protein 1 (CAP-1) and Toll-like receptor 4 (TLR-4) on cancer cells, leading to refractory responses towards GEM treatments [163] (Figure 2). Hence, GEM is more effective in macrophage-depleted mice than in their untreated counterparts [149]. On the other hand, simvastatin mitigates TAM-mediated GEM resistance by attenuating the TGF-β1/growth factor independence-1 (Gfi-1) signaling axis. Molecular studies elucidated that simvastatin reverted the TAM-mediated and TGF-β1-dependent downregulation of Gfi-1 and upregulation of connective tissue growth factor (CTGF), as well as high mobility group box 1 (HMGB1) that typically drives resistance to GEM [164]. CTGF was similarly identified as an important factor contributing to GEM resistance in animal models [165]. Simvastatin not only upregulates Gfi-1 expression, which increases the sensitivity towards GEM, but also suppresses TGF-β1 production that is released from TAMs [164]. Contrarily, Gabitova-Cornell et al. reported that statins can activate sterol regulatory element-binding protein 1 (SREBP1), which promotes TGF-β1 expression followed by epithelial–mesenchymal transition (EMT), in genetically engineered mouse models directed by oncogenic mutant *KrasG12D* and homozygous null *P53* [166]. Their study suggests that statin treatment can promote the mesenchymal type of PDAC, leading to a worsened prognosis [166]. The discrepancies between the two studies may be partly attributed to mice xenograft models using pancreatic cancer cell lines [164] versus conditionally genetic knockout mice [166].

Moreover, following treatment with chemo- or radiotherapy, cancer cells release inflammatory molecules, including the chemokine CCL2 that further recruits macrophages and promotes tumor proliferation and vascularization [121,167]. Consequently, CCL2 attenuates the efficacy of FOLFIRINOX chemotherapy or radiotherapy in mice. Hence, commencing the blockade of CCL2 using antagonistic antibodies can prevent macrophage recruitment and restore the sensitivity of PDAC towards chemotherapeutic and radiotherapy treatments [121,167].

### 3.2. Carcinogenic Impact of TAM-Secreted Extracellular Vesicles (EVs) or Exosomes on PDAC Progression

Extracellular regulators governing cancer progression can be encapsulated in a cargo-like structure collectively known as EV. They are released particles with variable sizes, ranging from 30 to 120 nm (named as exosomes) or 100 to 1000 nm (classified as microparticles) and generated from cell-derived membrane vesicles, and are enclosed within a phospholipid bilayer structure, although they are not proliferative [168,169]. They play pivotal roles in mediating intercellular communication under both physiological and pathological conditions [170,171] by disseminating genetic materials, proteins, metabolites, cancer regulators, or chemoresistant traits to neighboring cells through cellular internalization [172,173]. miRNAs contained in macrophage-derived exosomes (MDEs) can be transferred from TAMs to PDAC cells, resulting in altered gene expression and behaviors. For instance, the dislodging of miR-365 shed from MDEs abolished GEM efficacy through the upregulation of the triphosphonucleotide pool and the induction of CDA in cancer cells [174] (Figure 2).

Through EVs, TAMs communicate with malignant cells to orchestrate carcinogenic progression as well as chemoresistance [175]. In this regard, Xavier et al. carried out proteomic analysis and identified chitinase 3-like-1 (CHI3L1) and fibronectin 1 (FN1) as being the two most abundant proteins in the cargo of TAM-released EVs that play important roles in boosting GEM resistance in PDAC [176] (Figure 2). Further bioinformatics predictions using the cancer genome analysis (TCGA) supported this notion and revealed excessively expressed CHI3L1 and FN1 are associated with low OS in PDAC patients and high abundance of TAMs [176]. CHI3L1 is a secreted glycoprotein and a binding member of the mammalian chitinase-like proteins involved in various disorders, including cancer [177]. Several studies have indicated high expression of CHI3L1 with tumor grade, unfavorable prognosis, and metastasis in various human cancer types [178,179,180]. Through activating ERK signaling, CHI3L1 is not only partially responsible for GEM resistance in PDAC [176] (Figure 2), but is also similarly associated with chemoresistance towards other agents like paclitaxel and bevacizumab in ovarian as well as gastric cancers [178,181,182]. In light of FN1, recent studies demonstrated that increased FN1 secretion by PDAC stellate cells is correlated with aggressive tumor characteristics [183,184] and promotes GEM resistance through the same ERK pathway [185] (Figure 2). Together, a corroborative study carried out via the ectopic overexpression of CHI3L1 and FN1 using recombinant human proteins led to ameliorated GEM resistance [176]. Using a reciprocal approach by implementing CHI3L1 and FN1 inhibitors resulted in partial sensitivity restoration and improved treatment outcomes [176].

### 3.3. TAMs Promote Cancer Growth

TAMs are known to promote cancer growth and metastasis by secreting various factors [186,187]. IL-1β, a potent and versatile cytokine released from TAMs, plays a pivotal role in cancer cell proliferation, neoplastic progression, and metastasis. The activation of IL-1β requires an inflammasome, a multimeric cytosolic protein complex that assembles in response to cellular perturbations [188] (Figure 2). Nucleotide-binding and leucine-rich repeat receptor containing pyrin domain 3 (NLRP3)-induced inflammasomes in TAMs can activate IL-1β and macrophage polarization [189]. Through Gene Expression Omnibus public database analysis and implementing a set of in vitro and in vivo experiments, Gu et al. elucidated the effects of NLRP3 activation on TAM polarization and the subsequent lung metastasis in a mouse model of PDAC [189]. Conversely, NLRP3 depletion resulted in the opposite effects in colorectal carcinoma [190], gastric [191], prostate [192], and breast cancer [193]. Reciprocally, PDAC-derived cell debris can augment IL-1β production from M2 macrophages via crosstalk between the Toll-like receptor 4 (TLR4)/TRIF/NF-κB and FcγRI/III-SYK signaling pathways, and this effect can be boosted by IgG in PDAC cells. Not only enhancing cancer cell proliferation, upregulated IL-1β expression results in an immunosuppressive TME, promotes the EMT of malignant cells, invasion, and the subsequent metastasis [194]. 

By signaling through oncogenic mutant KRAS, PI3K, and p38 MAPK pathways, Bcl-2-associated athanogene 3 (BAG3) can be released from PDAC cells and then activate macrophages through its binding to a specific receptor named Interferon-Induced Transmembrane Protein 2 (IFITM-2) [195] (Figure 1). In this paracrine manner, BAG3-activated TAMs produce factors that conversely stimulate and amplify PDAC proliferation [152]. Treatment with inhibitory BAG3 antibody resulted in tumor regression and metastatic inhibition in three independent mouse models [195]. Consistently, delivering the humanized anti-BAG3 antibody BAG3-H2L4 abrogates this binding and leads to a prominent growth reduction in the Mia PaCa-2 pancreatic cancer cell xenograft model [196]. BAG3 is constitutively expressed in several primary tumors and tumor cell lines, including PDAC, where it plays a prosurvival role through various mechanisms according to the cellular context [197,198,199]. Studies on 346 PDAC biopsies demonstrated that all of them expressed BAG3 intracellularly and survival was significantly shorter in patients with high BAG3 expression than in those with low BAG3 expression [200].

### 3.4. TAMs Exploit Immunosuppressive and Tumor-Supportive Milieu

Programmed death receptor-1 (PD-1), one of the well-characterized immune checkpoint modulators and the major target of ICB, is mainly expressed by CD8^+^ cytotoxic T lymphocytes, and its binding to ligands (PD-L1 or PD-L2 released from cancer cells, for example), hinders T cell proliferation, impairs intrinsic tumoricidal functions, and promotes T cell exhaustion [201,202]. Aberrant PD-L1 expression by malignant cells has been identified in several solid cancer types and, therefore, comprises an important immune evasion mechanism [203]. High levels of PD-L1 indicate poor OS [204], and elevated PD-L1 expression is involved in immune escape rendering poor prognosis in triple-negative breast cancer [205]. Hence, by boosting the interactions among immune checkpoint modulators, TAMs protect malignant cells from being destroyed by antitumor T cells [206,207]. TAMs upregulate the expression of PD-L1 from cancer cells that consequently bind immune-suppressive receptors on T cells, resulting in impaired tumoricidal ability, proliferation, and effector functions [208]. For instance, tumor necrosis factor (TNF)-α can be released from TAMs, thereby upregulating PD-L1 expression in PDAC cells through NF-κB signaling, and this effect can be attenuated by neutralizing anti-TNF-α antibodies [209] (Figure 2). An in vitro study by cocultivating PDAC cells with TAMs recapitulated the same phenomenon. Clinically, elevated PD-L1 expression was not only positively correlated with macrophage infiltration, but also significantly associated with poor prognosis in 235 PDAC patients [209].

TAM-derived TGF-β1 is a well-established modulator that hinders tumor response towards PD-L1 blockade therapy [210] through the elevated expression of PD-L1 in PDAC cells [211]. TGF-β1 not only induces the nuclear translocation of pyruvate kinase isoform M2 (PKM2), but also promotes interaction between PKM2 and STAT1, leading to the transcriptional activation of PD-L1, owing to the concomitant binding of PKM2 and STAT1 to the PD-L1 promoter [211] (Figure 2). Hence, PKM2 knockdown decreases PD-L1 expression in PDAC cells and suppresses tumor growth. Moreover, the combination of PD-1/PD-L1 blockade along with PKM2 knockdown synergizes tumor regression [211]. Another independent study ratified the important role played by PKM2 in being a coactivator of HIF-1α, highlighting the crucial roles played by PKM2 through augmenting the expression of PD-L1 and contributing to cancer growth under a hypoxic TME [212] (Figure 2). Moreover, the immune checkpoint ligand Dectin-1 is highly released from TAMs in mouse and human PDAC [92]. The binding of Dectin-1 to its receptor gal-9 expressed on infiltrating immune cells results in tolerogenic programming and the masking of immune recognition, thereby favoring malignant cell growth [92] (Figure 2). Thus, dual treatments encompassing the depletion of TAMs and implementing ICB agents to abolish immune checkpoints can revive tumoricidal effects [213,214]. Moreover, cytokines or chemokines, such as CCL22, CCL28, CXCL12, CCL5, and CCL1, produced from TAMs can hamper the recruitment of CD8^+^ T lymphocytes [215,216] (Figure 2). Conversely, eradicating proliferating TAMs can improve T cell infiltration and promote their spatial redistribution proximally towards tumor cores, thereby rendering favorable treatment outcomes [75].

TAMs from human and murine PDAC are known for their high expression of apolipoprotein E (ApoE) that further upregulates CXCL1 and CXCL5, the chemokines known to impair tumoricidal T cell infiltration in PDAC and then launch an immunosuppressive TME [57,59,217,218,219] (Figure 2). The stimulation of these chemokines is mediated through low-density-lipoprotein receptors (LDLRs) and the NF-κB signaling axis [57] (Figure 2). Gene set enrichment analysis (GSEA) elucidated that the treatment of tumor cells with recombinant ApoE upregulates NF-κB signaling, which in turn augments the expression of CXCL1 and CXCL5 acting as chemoattractants for immune-repressive myeloid cells [57]. These recruited immune cells cause a sluggish infiltration of tumoricidal CD8^+^ T lymphocytes in PDAC [59,217,219]. Conversely, tumors evolved from ApoE knockout (ApoE^−/−^) mice have reduced cancer growth, lowered tumor burden, lessened fibrosis, and fewer immune-suppressive cells (Treg and MDSC), while also having increased cytotoxic CD8^+^ T lymphocyte infiltration. Apart from releasing chemokines, TAMs can dampen T lymphocytes’ anticancer actions by generating metabolic mediators. The metabolism and consumption of L-arginine or L-tryptophan by TAMs decrease the expression of the CD3ζ chain on T cells, resulting in T cell anergy and proliferation arrest [220,221,222] (Figure 2). Similarly, increased arginase I production by TAMs disrupts T cells’ metabolism and disables their cytotoxic effects against cancer cells [223].

### 3.5. TAMs Augment EMT, Invasion, Migration, Angiogenesis, Metastasis, and Lymphangiogenesis

TAMs are the major type of immune cells that participate in various aspects of carcinogenesis, including paving the path to invasion, metastasis, angiogenesis, and treatment resistance. Among them, the EMT transforms epithelial cells into a spindle-like mesenchymal population, resulting in increased motility, invasiveness, metastasis, and acquisition of cancer stem-like cells. The EMT has drawn ample research attention due to its roles of enhancing metastasis and chemoresistance, the two leading causes of mortality for PDAC [224]. Upon coculturing with M2 macrophages, PDAC gains the abilities of cell proliferation and migration, as well as the upregulation of mesenchymal markers and concomitant downregulation of epithelial hallmarks [155]. TAM-secreted cytokines and chemokines, including IL-1β [194], CCL18 [225], and IL8 [226], are known to foster the EMT in PDAC through the TLR4/IL10 axis or protease-activated receptor (PAR)1 signaling pathways [155,227]. Likewise, TGF-β released from M2-macrophages can bind its receptors on the PDAC cell surface and trigger the phosphorylation of Smad2/3 that subsequently actives the phospho-Smad2/3/4 complex, leading to enhanced Snail transcription (Figure 2). Afterwards, Snail hinders E-cadherin expression, promotes the EMT shift, and potentiates PDAC metastasis [228]. Such effects can be abolished by blocking the TGF-β pathway or by introducing antagonistic TGF-β antibodies [228].

MMP-mediated ECM degradation plays an important role in cancer invasion [229]. By secreting MMP-9 and by promoting EMT, TAMs have unveiled versatile effects on exacerbating PDAC migration and invasion [155,227]. Moreover, the macrophage-derived proinflammatory chemokines, namely CC-chemokine ligand 20 (CCL20) and macrophage inflammatory protein-3α (MIP3α), all bind CC-chemokine receptor 6 (CCR6) on PDAC cells, leading to upregulated MMP-9 expression and tumor invasion [230,231,232,233] (Figure 2). Additionally, macrophage-derived CCL18 empowers the invasive property by enhancing VCAM-1 expression (Figure 2). In a paracrine manner, VCAM-1 promotes lactate production by pancreatic neoplastic cells and further augments the polarization of macrophages towards the M2 phenotype, thereby establishing a positive feedback circuit [234]. Consistent with the role of macrophages in supporting metastasis, the pharmacological depletion of TAMs in mice prohibits the spread of PDAC cells to the liver, lung, and spleen [149,235].

Complicated metastatic spreads can be fostered by communication and networking between the local TME, malignant cells, and the nearby organs. Initially, malignant cells gain the ability to pass through the basement membrane into the surrounding stroma, where they can then enter adjoining organs such as the liver, lung, and peritoneum [236]. Intravasation is the foremost step for dissemination, and cancer cells often accomplish this process with the assistance of other cell types. For example, proteinases released from TAMs destroy the basement membrane prior to cancer cell dissemination [104]. M2 macrophages can harness tumor cells to intravasate through the vessel wall. After intravasation, circulating neoplastic cells ought to extravasate through the vessel wall in an adjacent organ prior to paving a metastatic niche [237,238]. It is noteworthy to mention that when influenced by the conditioned media generated from PDAC cancer cells in vitro, TAMs gained prominent glycolytic activities responsible for the acquisition of the prometastatic phenotype. Hence, the inhibition of glycolysis in TAMs impedes their ability to promote tumor cell extravasation, EMT, and angiogenesis [239].

Macrophages prime the premetastatic niches by serving as a “landing blueprint” for the homing of circulating cancer cells [235,240]. In this regard, the uptake of PDAC-derived exosomes by the resident liver macrophages, for example, results in the activation of fibrotic pathways and the establishment of a proinflammatory milieu that fosters metastasis [241]. Exosomal constituents from PDAC promote the secretion of TGF-β by liver macrophages, which in turn ameliorates the deposition of fibronectin by hepatic stellate cells [241], followed by the recruitment of bone-marrow derived monocytes to the liver, leading to the formation of a premetastatic niche [241]. Furthermore, tumors require angiogenesis to supply nutritional and oxygen demands. TAMs augment angiogenesis through the secretion of vascular endothelial growth factor (VEGF) that further crosstalks with the oncogenic transcription factors HIF1α, NFκB, and STAT3. Collectively, they promote an angiogenic switch and enhance blood vessel formation for tumor expansion [235]. Clinical evidence ratifies this notion, as TAMs are highly abundant in hypoxic areas and their presence correlates with increased blood capillary density in not only PDAC [242,243,244] but also in breast carcinoma [245]. A subset of monocytes that express the receptor tyrosine kinase with immunoglobulin and epidermal growth factor homology domain-2 (TIE2) also exploit enhanced proangiogenic activity in PDAC via the binding to angiopoietins for promoting blood vessel formation [246,247,248]. Indeed, the positivity of TIE2^+^ monocytes and TAMs correlates with increased microvessel density and the vulnerability of developing metastatic dissemination of PDAC [248]. Moreover, M2-macrophage-derived exosomes (MDEs) increase vascular density and promote the growth of subcutaneous tumors in a mouse model [244]. Intriguingly, miR-155-5p and miR-221-5p levels in the MDEs of M2 are higher than those in their control counterparts [244].

Lysyl oxidase-like protein 2 (LOXL2) is known for its contribution towards cancer advancement and metastasis in various cancer entities including PDAC [154]. *Loxl2* loss significantly decreases metastasis and improves OS. This effect is largely attributed to non-cell autonomous constituents evolved from ECM remodeling. Reciprocally, *Loxl2* overexpression promotes cancer growth with lowered OS and poor prognostic outcomes, which is ascribed from enhanced EMT and the increased acquisition of cancer stem cells [154]. Further studies identify TAM-secreted oncostatin M (OSM) as being the activator for LOXL2 expression, and, therefore, abrogating macrophages can hinder *Osm* and *Loxl2* functionalities and diminish metastasis in mouse models [154] (Figure 2). Moreover, during PDAC progression, CCR2^+^ inflammatory monocytes are recruited to the liver through peripheral blood circulation, where they establish a metastatic niche [120]. Once in the liver, the effector macrophages secrete granulin that activates resident hepatic stellate cells to myofibroblasts. Subsequently, myofibroblasts secrete periostin that fosters a fibrotic TME and metastatic spread [249]. The disruption of CCR2 or the genetic ablation of granulin inhibits macrophage recruitment and protects against liver metastasis [120,249].

By embarking on regional lymph nodes, TAMs aid lymphangiogenesis that formulates a crucial route aggravating cancer cell dissemination. Clinical evidence reinforces the idea that high lymphatic density corresponds to increased lymph node metastasis and lowered OS in PDAC patients [250]. The molecular cue is exemplified in Figure 2 that lymphangiogenesis is regulated by the binding of VEGF-C, a ligand overexpressed by cancer cells, to its target receptor VEGFR-3 on TAMs. This complex then favors lymphangiogenesis via activating lymphatic endothelial cells [251] or by secreting lymphangiogenesis-promoting factors, including VEGF and MMP-9 [252]. 

### 3.6. Impact of TAMs on the Acquisition of Pancreatic Cancer Stem-like Cells (PCSCs)

PCSCs are the unique subfraction of pleiotropic cancer cells that can self-renew and then differentiate to heterogeneous lineages [253]. Thus, they play versatile roles in tumor progression, metastasis, and chemoresistance [254]. TAMs provide compelling signals to acquire and sustain PCSCs. Clinical studies demonstrated that the abundance of TAMs correlates with PCSC density in PDAC, and it is associated with a poor OS [65]. Although PCSCs can mold their own niche and maintain their self-renewing and tumorigenic properties, the TME also provides cues to support PCSCs. In this regard, TAMs play the pivotal roles of potentiating and empowering the acquisition of PCSCs. By expressing CD51, that subsequently activates the paracrine TGF-β1/smad2/3 signaling pathway and enhances the expression of stemness-related transcription factors like Nanog, Sox2 and Oct4, TAMs augment the formation of PCSCs [255,256] (Figure 2). Suppressing CD51 expression in macrophages effectively diminishes PCSCs, suggesting that abrogating CD51 can be developed into an innovative promising therapeutic modality [256]. 

The intricate crosstalk between PCSCs and TAMs renders an important driver for tumor development in PDAC. In response to IFNγ released from PCSCs, TAMs secrete IFN-stimulated gene 15 (ISG15), which subsequently enhances PCSC phenotypes in PDAC in vitro and in vivo. This circuit thereby reinforces the positive feedback loop amplification and self-renewal of PCSCs, invasive capacity, and tumorigenic potential [257]. Apart from ISG15, TAMs also release immunomodulatory cationic antimicrobial peptide 18/LL-37 (hCAP-18/LL-37) that consequently activates PCSCs, promotes cancer growth, EMT, and metastasis [258] (Figure 2). As a return, PCSCs act as the major supplier of the TGF superfamily members Nodal/Activin A and TGF-β1, which induce the additional polarization of M2 [259]. 

## 4. Conclusions

It has been widely acknowledged that the sophisticated interplay between cancer and immune surveillance determines whether neoplastic cells will survive or be eradicated. The battle between tumoricidal and tumor-promoting activities relies on the tumor microenvironment niche. PDAC has been recognized as a “cold” malignancy due to the scanty infiltration of cytotoxic CD8^+^ T lymphocytes or dendritic cells that ought to penetrate through the stiff desmoplastic stroma shielding the cancer cores. Yet, poor clinical outcomes presented by the failures of ICB, and other immunotherapeutic treatments, elucidate that the PDAC microenvironmental niche has been hijacked and the evolved immune-suppressive TME is largely orchestrated by M2 macrophages, exacerbating neoplastic progression. This report attempts to shed a light on developing future promising regiments to eradicate this deadly cancer, partly by attenuating M2 macrophage polarization, hindering neoplastic growth and metastasis, deteriorating desmoplastic stroma, reverting the immune-suppressive tumor microenvironment, diminishing pancreatic cancer stem-like cells, and enhancing susceptibility to GEM and immune checkpoint blockade therapies. Combinatorial treatment protocols may also include blocking oncogenic signaling from mutant KRAS and from other stromal constituents, correcting aberrant epigenomes, eliminating extracellular vesicles that typically promote carcinogenesis, diminishing the EMT to hinder the metastasis and acquisition of cancer stem-like cells, as well as boosting the infiltration of antitumor immune cells [260] (partly exemplified in Table 1).

## Figures and Tables

**Table 1:** Exemplified therapeutic targets for treating PDAC by maneuvering M2, PDAC, and the TME.

Agent	Target	Rationale	Reference(s)
Inhibitor clodronate liposomes	Proliferating TAMs	TAMs suppress CD8^+^ T lymphocytes and provoke chemoresistance	[75,149,161]
Exosomes containing siRNA that abrogates gal-9	PDAC expressing gal-9	The binding of gal-9 to dectin-1 on macrophages promotes M2 polarization	[92,93,94]
Antagonist miR-155 and miR-125b2	Macrophage polarization	These miRs favor macrophage polarization toward M1	[99]
Inhibitor BLZ945	Block CSF1R on macrophages	The binding of CSF to CSF1R ameliorates M2 polarization	[102]
Inhibitor pomalidomide	Irf4	Irf4 supports M2 polarization	[111]
Antagonistic αIL-20 Ab	IL-20	IL-20 promotes M2 polarization	[112]
Inhibitor SRX3207	SYK and PI3K	Both signal transducers enhance M2 polarization	[116]
Inhibitor PF-04136309	CCR2	CCL2-CCR2 axis supports the recruitment of monocytes from bone marrow to the tumor bed	[121]
Agonistic αCD40 Ab selicrelumab	CD40	Activated CD40 favors M1 polarization and restores immune surveillance	[126,127]
Inhibitor PT2399	HIF-2	HIF-2 improves the recruitment of M2 macrophages	[143]
Inhibitor metavert	HDACs and GSK3	These regulators enhance M2 polarization and chemoresistance	[147]
Inhibitor simvastatin	Suppress TGF-β1/Gfi-1 signaling	This signaling pathway fortifies chemoresistance	[164]
Antagonistic αCCL2 Ab	CCL2	CCL2-CCR2 axis strengthens the recruitment of monocytes to the tumor bed	[167]
Inhibitor pentoxifylline	CHI3L1	CHI3L1 in MDEs enhances GEM resistance	[176]
Inhibitor pirfenidone	FN-1	FN1 in MDEs augments GEM resistance	[176]
Antagonistic BAG3-H2L4 Ab	BAG3	BAG3 released from PDAC activates TAMs	[196]
Antagonistic αTNF-α Ab	TNF-α	TNF-α from TAMs upregulates PD-L1 in PDAC	[209]
Antagonistic αTGF-β Ab	TGF-β	TGF-β released from M2 promotes EMT	[228]
